# Preventing Cisplatin-Induced Neuropathy and Related Emotional Disorders with the Coadministration of Duloxetine and Hydrogen-Rich Water in Male and Female Mice

**DOI:** 10.3390/antiox14081004

**Published:** 2025-08-16

**Authors:** Ignacio Martínez-Martel, Sylmara Esther Negrini-Ferrari, Olga Pol

**Affiliations:** 1 Grup de Neurofarmacologia Molecular, Institut de Recerca Sant Pau (IR SANT PAU), Sant Quintí 77-79, 08041 Barcelona, Spain; 2 Grup de Neurofarmacologia Molecular, Institut de Neurociències, Universitat Autònoma de Barcelona, 08193 Barcelona, Spain

**Keywords:** anxiety, chemotherapy-induced peripheral neuropathy, cisplatin, depression, duloxetine, hydrogen-rich water, inflammation, molecular hydrogen, oxidative stress

## Abstract

Cisplatin (CIS)-induced peripheral neuropathy and associated comorbidities have a detrimental effect on the lives of cancer patients. Currently, there are no effective therapies to alleviate these symptoms. Duloxetine (DULO) is a recommended treatment, but it is linked with important side effects, thus making it essential to explore novel approaches. We examined the impact of a prophylactic treatment with a low dose of DULO combined with hydrogen-rich water (HRW) on CIS-injected C57BL/6 male and female mice as a possible therapy for allodynia, muscle and body weight deficits, and emotive syndromes accompanying this type of chemotherapy. The prophylactic treatment with DULO and HRW prevented mechanical allodynia caused by CIS in both sexes and had greater effects than either treatment given individually. The combined treatment also prevented cold allodynia in male mice but only reduced it in females. Moreover, the coadministration of DULO with HRW avoided muscular deficits in both sexes. Furthermore, the body weight reduction induced by CIS in both sexes was not entirely mitigated by the combined therapy. However, all treatments avoided the anxiety- and depressive-like behaviors elicited by CIS. The antiallodynic actions and prevention of muscular deficits produced by the combined treatment might be explained by the inhibition of oxidative stress, inflammatory responses, and plasticity alterations provoked by CIS in the dorsal root ganglia of these subjects. This study proposes, for the first time, the cotreatment of DULO with HRW as an effective therapy for CIS-induced peripheral neuropathy and reveals the influence of sex on these actions.

## 1. Introduction

Cancer is the second leading cause of the global burden of mortality (World Health Organization, 2022), responsible for approximately one out of six cases of human worldwide deaths [1]. Despite advances in chemotherapy types and regimens, a sizable proportion of patients fail to adhere to chemotherapy or tend to quit treatment, mainly because of remarkable chemotherapy-related side effects [2].

Cisplatin (CIS), a first-generation platinum-based chemotherapeutic agent, is prescribed for the treatment of several solid tumor types, for instance, colorectal cancer, lung, ovarian, bladder, breast, prostate, melanoma, and testicular cancers [3]. However, despite its antitumoral effectivity, the peripheral neuropathy and emotional disorders associated with CIS are significant side effects of this therapy that affect 30% to 35% of patients, decreasing their quality of life [1,4].

Currently, there are no effective treatments available to alleviate neuropathic pain caused by chemotherapy. The American Society of Clinical Oncology’s (ASCO) clinical practice guidelines issued only a moderate recommendation for the antidepressant duloxetine (DULO). However, high accumulative doses of this drug produced substantial side effects, including intestinal, hepatic, and cardiovascular alterations and/or anxiety, seizures, and somnolence [5,6]. Therefore, it is imperative to explore novel strategies to enhance the efficacy of DULO and mitigate its adverse effects.

Several preclinical studies have demonstrated the antiallodynic effects of DULO. For example, in animals with neuropathic pain induced by chemotherapy, the single administration of 30 mg/kg of DULO demonstrated efficacy against mechanical and thermal allodynia; conversely, single doses of 10 mg/kg were effective only in combination with other drugs, for example, minocycline [7]. Regarding the repetitive administration of DULO, animal studies evidenced that lower doses, such as 5 mg/kg, were able only to moderately revert allodynia [8,9].

Another approach to inhibiting chemotherapy-induced peripheral neuropathy involves molecular hydrogen, a gas with protective properties and no demonstrable relevant secondary effects, characterized by its high capacity to cross the blood–brain barrier and penetrate cell membranes [10]. As a result, molecular hydrogen has proven to be an optimal candidate for reducing the nociceptive responses and the anxiogenic and depressant-related behaviors linked with nerve-injury-induced neuropathic pain [11]. These effects are related to its capacity to reduce inflammation and protect cells from damage by blocking the production of NF-κB and the NLRP3 inflammasome, as well as by modulating the oxidant and antioxidant systems [12,13,14]. Moreover, recent experiments further revealed that the administration of hydrogen-rich water (HRW) at 0.3 mM had beneficial effects in animals with paclitaxel-induced neuropathic pain [15]. Considering these data, the use of low doses of DULO in combination with a low concentration of HRW seems to be a promising tool for minimizing the adverse effects produced by high doses of DULO while achieving similar or even greater therapeutic effects.

The pathogenesis of CIS-induced peripheral neuropathy is related to abundant biochemical alterations involving inflammation and oxidative stress. CIS injection, like other antineoplastic drugs such as paclitaxel, vincristine, and oxaliplatin, activates the NLRP3 inflammasome, which in turn promotes the synthesis of proinflammatory cytokines, resulting in neuropathic pain and muscle wasting [16].

Recent research has supported the idea that oxidative stress is another critical mechanism underlying the onset and progression of chemotherapy-induced neuropathic pain. Indeed, altered lipid peroxidation, reflected in elevated levels of 4-hydroxynonenal (4-HNE) with consequent exacerbation of oxidative stress [17], and decreased activity of the antioxidant enzymes superoxide dismutase 1 (SOD-1) and heme oxygenase 1 (HO-1) in the dorsal root ganglia (DRG) and prefrontal cortex (PFC) of CIS injected-mice have been demonstrated [18].

Other studies have highlighted the involvement of MAPK signaling pathways in the development and progression of chemotherapy-induced peripheral neuropathy. In response to oxidative stress, CIS activates JNK phosphorylation (p-JNK), which in turn promotes the activation of proapoptotic Bcl-2 family proteins, triggering mitochondrial dysfunction and the release of cytochrome c, caspase activation, and neuronal apoptosis [19]. Therefore, elevated levels of p-JNK have been described in the DRG of animals with CIS-induced peripheral neuropathy [19].

Our current research aimed to assess the efficacy of a prophylactic treatment that combines a low dose of DULO (5 mg/kg) with a low concentration of HRW (0.15 mM) as a novel approach to protect mice from CIS-induced neuropathy, grip strength deficiencies, weight loss, and mental illnesses such as anxiety- and depressive-like behaviors. Additionally, we examined the impact of sex on these responses and on the principal molecular pathways involved in their development.

## 2. Materials and Methods

### 2.1. Animals

Male and female C57BL/6J mice at 10 weeks of age at the commencement of the experiments were used. Mice were obtained from Envigo Laboratories in Barcelona, Spain and kept in a 12 h dark/light cycle with an unlimited supply of food and water, a temperature of 22 °C, and a humidity level of 66%. Following a week of acclimation to the housing arrangements, the experimental procedure was carried out from 9:00 to 17:00 in compliance with the guidelines from the European Commission (2010/63/EC) and Spanish law (RD 53/2013) regulating animal research. The local Committee of Animal Use and Care of the Autonomous University of Barcelona approved these experiments (CEEA-UAB-protocol # 4581). Four male or female mice were housed in polypropylene cages, which were equipped with a carton hut and cellulose fragments to facilitate an enriching environment. Male and female mice were randomly assigned to each experimental group. Random numbers were generated using the standard = RAND ( ) function in Microsoft Excel. In this study, we used a total of 160 mice (80 male and 80 female).

Behavioral testing was conducted between 9:00 a.m. and 5:00 p.m. For each test, the order of animals being tested was randomized daily, and each animal was tested at a different time on each test day. For each animal, two different investigators participated as follows. A first investigator administered the treatment according to the randomization table. This investigator was the only one aware of the treatment group assignment. A second investigator, who was blinded to the treatment, assessed the animals’ mechanical and thermal allodynia, grip strength deficits, and weight loss. A similar strategy was used for evaluating the effects of treatments on the anxiety- and depressive-like behaviors caused by CIS.

### 2.2. Experimental Design

**Figure 1:**
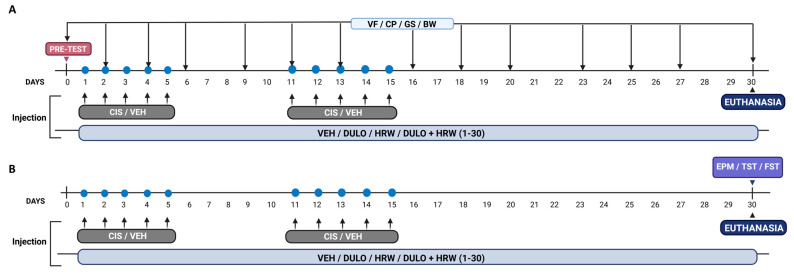
Graphical depiction of the experimental timeline. The experiments for evaluating the effects of DULO combined with HRW on the mechanical and cold allodynia and the grip strength and weight loss instigated by CIS (**A**) and on the development of emotional disorders associated with CIS-induced chemotherapy (**B**) in male and female mice are represented. VF, von Frey; CP, cold plate; GS, grip strength; BW, body weight; EPM, elevated plus maze; TST, tail suspension test; FST, forced swimming test; CIS, cisplatin; VEH, vehicle; DULO, duloxetine; HRW, hydrogen-rich water.

**Figure 2:**
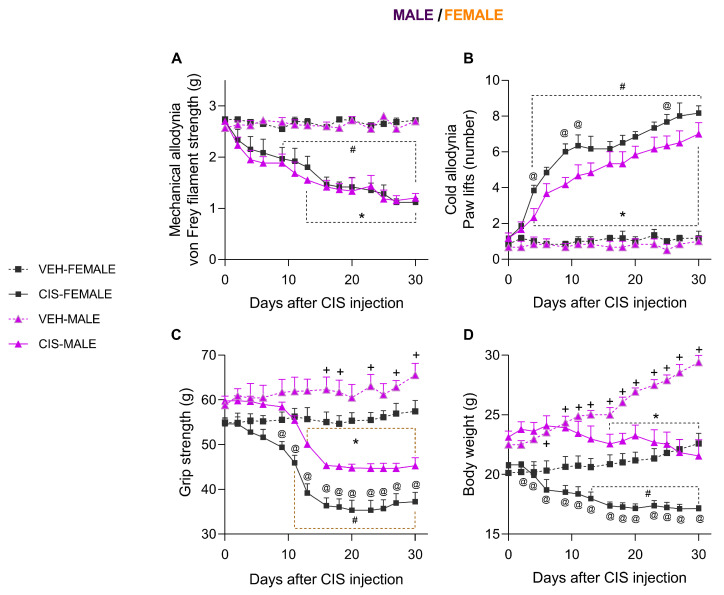
CIS injection provoked mechanical allodynia, cold allodynia, grip strength deficit, and body weight loos in male and female mice. The mechanical allodynia (**A**) and cold allodynia (**B**) on the hind paws and the loss of grip strength (**C**) and body weight (**D**) incited by CIS in male and female animals are represented. In all panels, each symbol indicates significant differences between * CIS-MALE and VEH-MALE, # CIS-FEMALE and VEH-FEMALE, + VEH-MALE and VEH-FEMALE, and @ CIS-FEMALE and CIS-MALE mice (*p* < 0.05, one-way ANOVA plus Holm–Šídák multiple comparisons test). Mean values ± SEM; n = 8 animals.

**Figure 3:**
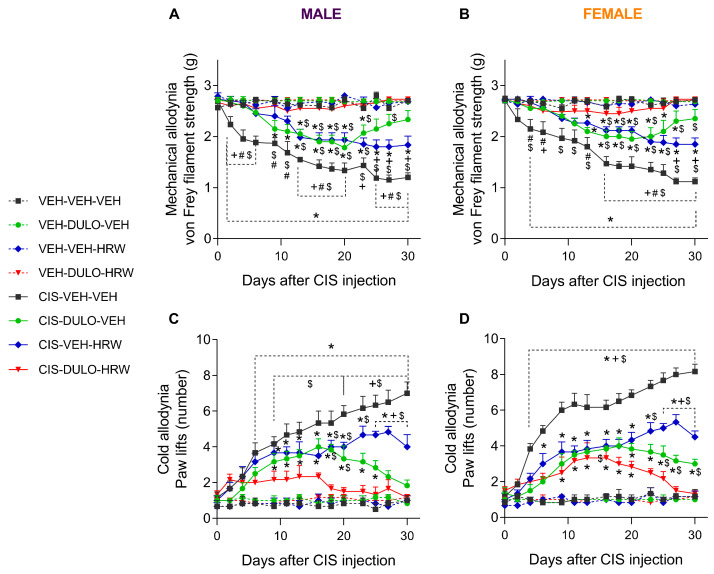
Treatment with DULO, HRW, and DULO plus HRW inhibited the mechanical and cold allodynia provoked by CIS in male and female mice. The effects of the prophylactic administration of DULO, HRW, and DULO plus HRW on the mechanical and cold allodynia observed on the hind paws of CIS-injected male (**A**,**C**) and female (**B**,**D**) mice are represented. In all panels, for each day assessed, * indicates significant differences vs. corresponding mice treated with VEH-VEH-VEH, VEH-DULO-VEH, or VEH-VEH-HRW; +, CIS-DULO-VEH; #, CIS-VEH-HRW; and $, CIS-DULO-HRW (*p* < 0.05, one-way ANOVA plus Holm–Šídák multiple comparisons test). Mean values ± SEM; n = 8 animals.

In different groups of male and female mice, we evaluated the impact of treatments with DULO (5 mg/kg) and HRW (0.15 mM) alone and in combination on the following: (1) tactile and cold allodynia, grip strength deficits, and body weight loss induced by intraperitoneal administration of CIS or VEH on days 2, 4, 6, 9, 11, 13, 16, 18, 20, 23, 25, 27, and 30 after injection (n = 8 animals) (Figure 1A); (2) the anxiety- and depressive-related behaviors generated by CIS at 30 days after the first injection (n = 8 animals) (Figure 1B); (3) the protein levels of 4-HNE, NLRP3, p-JNK, HO-1, SOD-1, and NAD(P)H: quinona oxidorreductasa 1 (NQO1) in the DRG and amygdala (AMG) on day 30 after the first CIS or vehicle (VEH) injection by Western blot analysis (n = 4–5 samples for group).

The sample size calculation was performed using the G* Power 3.1.7 software. Based on the results obtained in a pilot test and accepting a risk of alpha = 0.05 and beta = 0.2 in a two-tailed test, eight animals per group were needed to recognize as statistically significant the differences between groups. Every effort was made to reduce animal suffering and use the fewest possible animals. To minimize the number of animals used, tissues from animals subjected to the behavioral tests were used to perform the Western blot assays.

### 2.3. Administration of Drugs

CIS (Sigma Aldrich, St. Louis, MO, USA) was weakened in sterile saline (0.9% NaCl) and given intraperitoneally once a day for five days at a dose of 2.8 mg/kg. After five days off, the doses were given again for a second time [20], comparable to that used in patients [21]. A hydrogen water generator (Osmo-star Soriano S.L., Alicante, Spain) was employed to produce HRW from hydrogen, and DULO was acquired from Sigma Aldrich (St. Louis, MO, USA). The two compounds were given intraperitoneally alone or in combination once a day for 30 days, at a concentration of 5 mg/kg (DULO) and 0.15 mM (HRW) in a volume of 10 mL/kg, one hour before the tests [1,22]. Considering that our objective was to potentiate the analgesic actions produced by a low dose of DULO, and that a recent study revealed that the repetitive administration of HRW at 0.3 mM avoided the development of the allodynia provoked by CIS [23], we used a lower concentration of HRW (0.15 mM) that produces lesser antinociceptive effects (previously tested in a pilot study) to evaluate if it could potentiate the actions of DULO in animals with CIS-induced neuropathy.

For each group that was given a drug, an equivalent volume of an analogous VEH was injected to its corresponding control group.

### 2.4. Allodynia

Mechanical allodynia was quantified by measuring the hind paw withdrawal response to von Frey hair stimulation using the up-and-down method [24]. Mice were positioned in methacrylate cylinders (20 cm high/9 cm diameter) in a grid bottom. A series of von Frey hairs (0.4 to 3 g; North Coast Medical, Inc., San Jose, CA, USA) were perpendicularly applied to the plantar surface of each hind paw. Paw withdrawal or shaking was considered a positive response. The next lower hair was applied whenever there was a positive reaction, and the next higher hair was applied whenever there was a negative reaction.

We utilized a cold plate analgesiometer (Ugo Basile, Varese, Italy) to measure cold allodynia at a temperature of 4 ± 0.5 °C. The number of elevations of each hind paw was recorded for a period of 5 min.

### 2.5. Grip Strength Test

Grip strength, used to reflect muscle weakness, was measured employing a grip strength meter (Ugo Basile, Varese, Italy). The mice were allowed to grip the metal bar with both hind paws before their tail was gently pulled back. The maximal force (g) was recorded automatically by the apparatus [25]. The test was accomplished three times for each animal at 1 min intervals before CIS or VEH injection. This value was considered as 100% grip strength and was used as a reference for following determinations.

### 2.6. Anxiety- and Depressive-like Comportments

The elevated plus maze (EPM) and open field (OF) tests were employed to evaluate anxiety-like behaviors. The EPM consists of four arms, two of which are enclosed by 15 cm high walls and two of which are open, and is elevated 45 cm from the floor [26]. The arms are 35 cm long and 5 cm wide. Mice were permitted to investigate the maze for 5 min after being positioned in its center, and a digital camera was used to capture their behavior during this period. For each animal, the number of entries in the open and closed arms, as well as the time spent in the open arms, were written down.

In the OF test, mice were placed in the center of the arena of a 44 × 44 cm box with a grey, nonreflecting base and four walls, and their behavior was recorded by a digital camera for 5 min. Animals were allowed to move freely around the maze and to explore the environment. The number of entries in the central area, the time spent in it, and the number of squares crossed were assessed [27].

To evaluate depressive-like behaviors, the forced swimming test (FST) and the tail suspension test (TST) were applied. In the TST test, adhesive tape was applied to the tips of the mice’s tails, and they were secured to a surface suspended 35 cm above the ground. The animals were videotaped, and the time they remained immobile was measured for 6 min [28]. In the TST, mice were positioned in transparent cylinders (25 cm in height and 10 cm in radius) that contained water (24 ± 2 °C) to a depth of 10 cm for 6 min. The time that the animals remained immobile during the final 4 min was recorded [29].

Animals were adapted to the testing room for 1 h before beginning the tests.

### 2.7. Western Blot

On day 30 after the first injection of CIS or VEH, animals were euthanized by cervical dislocation. The DRG from the lumbar section (L3 to L5) and AMG were extracted and sonicated with cold RIPA buffer (Sigma-Aldrich, MO, USA). After solubilization for 1 h at 4 °C, the mixture was sonicated one more time for 10 s and centrifuged for 20 min at 700× g and 4 °C. Total protein samples (60 µg) were separated by 12% sodium dodecyl sulfate polyacrylamide gel electrophoresis and then transferred to polyvinylidene fluoride membranes for 2 h and blocked with phosphate-buffered saline plus Tween 20 or Tris-buffered saline plus Tween 20 + 5% of nonfat dry milk or bovine serum albumin for 75 min. The membranes were incubated first with specific primary antibodies overnight at 4 °C (Table 1) and subsequently with a horseradish peroxidase-conjugated secondary antibody (GE Healthcare, Little Chalfont, UK) for 1 h at room temperature. Immunohistochemistry was detected by chemiluminescence (ECL kit; GE Healthcare, Little Chalfont, UK) and quantified by densitometry using the Image-J program (version 1.8.0; National Institutes of Health, Bethesda, MD, USA).

### 2.8. Statistical Analyses

The software programs Prism 8.0 (GraphPad, La Jolla, CA, USA) and SPSS (version 28, IBM, Madrid, Spain) were used to conduct the statistical analysis. The data are presented as the mean values with the standard error of the mean (SEM). Three-way repeated measures ANOVAs with injection, sex, and time as the variation factors, followed by a one-way ANOVA and a Holm–Šídák multiple comparisons test, were used to assess the impact of sex on the mechanical and cold allodynia and the grip strength and body weight loss caused by CIS in mice. The effects of DULO combined with HRW on the mechanical and cold allodynia, as well as the grip strength and body weight deficits, triggered by CIS in male and female mice were analyzed using three-way repeated measures ANOVAs with treatment, sex, and time as the variation factors, followed by a one-way ANOVA and a Holm–Šídák multiple comparisons test.

Three-way ANOVA with injection, sex, and treatment serving as the variation factors were applied to examine the effects of treatment with DULO and HRW alone and in combination on the emotional disorders related to CIS-induced chemotherapy. These were followed by a one-way ANOVA and a Holm–Šídák multiple comparisons test. Finally, the capability of these treatments to mitigate the oxidative stress, inflammatory responses, and plasticity changes caused by CIS in the DRG and/or AMG of male and female mice was assessed through a two-way ANOVA with sex and treatment as the variation factors, followed by a one-way ANOVA and a Holm–Šídák multiple comparisons test. A value of *p* < 0.05 was considered significant.

## 3. Results

### 3.1. The Impact of Sex on the Mechanical and Cold Allodynia and the Grip Strength and Body Weight Loss Caused by CIS in Mice

Our outcomes confirmed that CIS similarly reduced the threshold of hind paw withdrawal due to von Frey filament stimulation from days 9 and 13 to 30 after injection in female and male mice, respectively (*p* < 0.001, one-way ANOVA vs. the corresponding VEH-injected mice; Figure 2A). That is, the three-way repeated measures ANOVA demonstrated significant effects of injection and time as well as and interaction between injection and time (Table 2). No significant differences between sexes were detected in VEH- or CIS-injected mice.

Concerning thermal allodynia, the three-way repeated measures ANOVA demonstrated significant effects of injection, sex, and time, as well an interaction between injection and time (Table 2). Accordingly, the augmented quantity of hind paw lifts instigated by cold stimulus in CIS-injected male mice from days 4 to 30 after injection (*p* < 0.001, one-way ANOVA vs. corresponding VEH-injected mice; Figure 2B) was increased in female mice at days 4, 9, 11, and 25 after CIS injection (*p* < 0.001, one-way ANOVA). The numbers of hind paw lifts observed in CIS-injected female animals were also different from those observed in their corresponding VEH-injected mice from days 4 to 30 after injection (*p* < 0.001, one-way ANOVA). No significant differences were observed between male and female mice injected with VEH.

Our data also verified the impact of sex on the grip strength deficits and body weight loss observed in CIS-injected mice. In both cases, significant effects of injection, sex, and time and an interaction between injection and time were observed. In reference to body weight, interactions between sex and time and among injection, sex, and time were also demonstrated (Table 2). The grip strength deficits caused by CIS in female mice from days 11 to 30 after its injection (*p* < 0.001; one-way ANOVA compared with their respective VEH-injected mice) were higher than those observed in CIS-injected male mice from days 9 to 30 after injection (*p* < 0.001; one-way ANOVA), which were in turn reduced as compared with VEH-injected male mice from days 13 to 30 (*p* < 0.001, one-way ANOVA; Figure 2C). Significant differences between male and female mice injected with VEH were detected from days 16 to 30 postinjection, where the grip strengths of VEH-injected male mice were higher than those of VEH-injected female mice. Comparable outcomes were identified in the loss of body weight incited by CIS in female and male mice from days 13 and 16 to 30 after injection, respectively (*p* < 0.001, one-way ANOVA compared with their respective VEH-injected mice; Figure 2D). Moreover, the loss of grip strength induced by CIS in female mice was greater than that observed in CIS-injected male mice from days 2 to 30 postinjection. Finally, significant differences between sexes were identified in VEH-injected animals, where the body weight of males was higher than that of females from days 6 to 30 after VEH injection (*p* < 0.001; one-way ANOVA).

### 3.2. The Effects of DULO Combined with HRW on the Mechanical and Cold Allodynia and the Grip Strength and Weight Loss Instigated by CIS in Male and Female Mice

Three-way ANOVA repeated measures showed significant effects of treatment, time, and their interaction on the inhibitory effects produced by the prophylactic administration of DULO and HRW alone and combined on the mechanical allodynia, cold allodynia, grip strength, and weigh loss caused by CIS in male and female mice (Table 3). Significant effects of sex were observed on thermal allodynia, grip strength, and body weight. Moreover, significant interactions between treatment and sex regarding thermal allodynia, as well as between sex and time and among treatment, sex, and time concerning body weight, were identified.

The results showed that in both sexes, the mechanical allodynia induced by CIS was partially and completely inhibited by the administration of DULO and HRW given alone and combined, respectively. Therefore, while DULO and HRW individually only partially inhibited mechanical allodynia from days 13 to 23 or 30 after CIS injection (*p* < 0.001; one-way ANOVA; vs. their corresponding VEH- and CIS-DULO-HRW-treated animals; Figure 3A), DULO plus HRW avoided the development of allodynia from 0 to 30 days of treatment in male mice. Moreover, whereas the effects of DULO were similar to those produced by DULO plus HRW at days 25 and 30 of treatment, the effects of HRW given alone were lower than those induced by DULO administered alone or combined with HRW from days 25 to 30 of treatment (*p* < 0.001; one-way ANOVA; Figure 3A).

**Figure 4:**
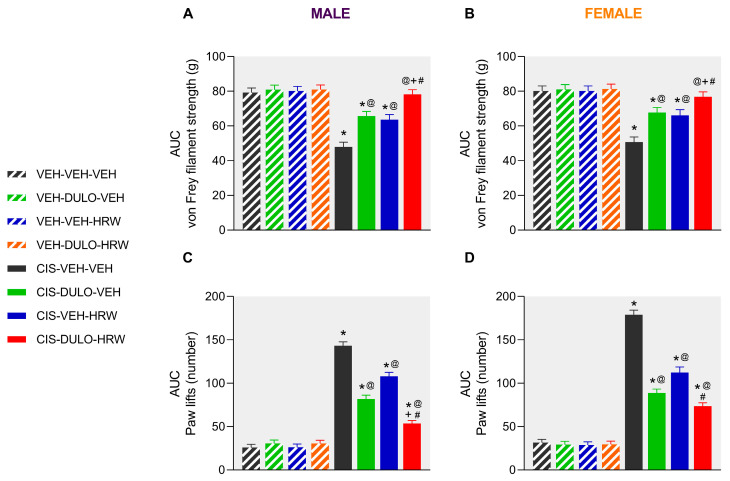
The global impact of DULO, HRW, and DULO plus HRW treatments on the suppression of the mechanical and cold allodynia induced by CIS. Data are presented as the AUC for the von Frey filament strength (g) and the number of hind paw lifts in the cold plate from male (**A**,**C**) and female (**B**,**D**) mice treated with DULO and HRW alone and in combination for 30 consecutive days. In all tests, * indicates significant differences vs. corresponding mice treated with VEH-VEH-VEH, VEH-DULO-VEH, or VEH-VEH-HRW; @, CIS-VEH-VEH; +, CIS-DULO-VEH; and #, CIS-VEH-HRW (*p* < 0.05, one-way ANOVA plus Holm–Šídák multiple comparisons test). Mean values ± SEM; n = 8 animals.

Similar effects were observed in female mice, but in this case, the partial inhibition of mechanical allodynia induced by DULO and HRW administered separately started at 16 days after CIS injection and remained inferior to that produced by its combination (*p* < 0.001; one-way ANOVA; Figure 3B). Furthermore, whereas the inhibitory effects of DULO were similar to those produced by DULO plus HRW at day 25 of treatment, the effects of HRW were lower than those induced by the combined treatment and DULO administered alone at days 27 and 30 of treatment (*p* < 0.001; one-way ANOVA; Figure 3B). The area under the curve (AUC) analysis supported the partial and full inhibition of mechanical allodynia provoked by DULO and HRW given alone and combined, respectively, in male (Figure 4A) and female mice (Figure 4B). It also showed that the effects of DULO plus HRW were stronger than the effects of each component given alone (*p* < 0.001; one-way ANOVA) (Table S1).

In both sexes, the combined administration of DULO plus HRW inhibited the cold allodynia caused by CIS, but this reversion was complete from days 0 to 30 of treatment in male mice (Figure 3C) but only partial from days 9 to 20 of treatment in female mice (*p* < 0.001; one-way ANOVA vs. VEH-VEH-VEH treated animals; Figure 3D). In both male and female animals, the antiallodynic actions produced by DULO given alone were higher than those made by HRW from days 25 to 30 of treatment (*p* < 0.001; one-way ANOVA). Moreover, while the prophylactic administration of DULO produced similar effects to those of DULO combined with HRW from days 23 to 30 of treatment in male mice (Figure 3C), the effects of the combined treatment were higher than those produced by DULO from days 25 to 30 of treatment in female mice (Figure 3D). Additionally, the AUC analysis supported that the combined treatment resulted in a greater inhibition of thermal allodynia than the individual administration of DULO and HRW in male (Figure 4C) and that of HRW in female mice (Figure 4D) (*p* < 0.001; one-way ANOVA) (Table S1).

**Figure 5:**
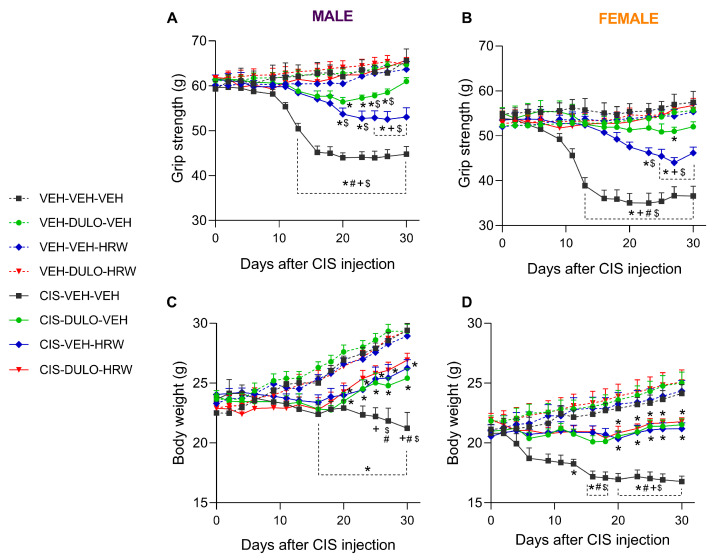
Treatment with DULO, HRW, and DULO plus HRW inhibited the grip strength deficits and body weight loss provoked by CIS in male and female mice. The effects of the prophylactic administration of DULO, HRW and DULO plus HRW on the grip strength deficits and body weight loss provoked by CIS in male (**A**,**C**) and female (**B**,**D**) mice are represented. In all panels, for each day assessed, * indicates significant differences vs. corresponding mice treated with VEH-VEH-VEH, VEH-DULO-VEH, or VEH-VEH-HRW; +, CIS-DULO-VEH; #, CIS-VEH-HRW; and $, CIS-DULO-HRW (*p* < 0.05, one-way ANOVA plus Holm–Šídák multiple comparisons test). Mean values ± SEM; n = 8 animals.

**Figure 6:**
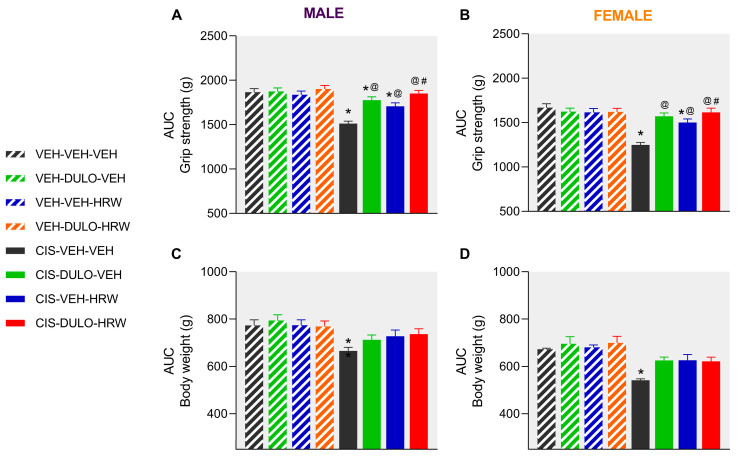
The global impact of DULO, HRW, and DULO plus HRW treatments on the inhibition of the grip strength deficits and body weight loss provoked by CIS in male and female mice. Data are presented as the AUC for the grip strength (g) and body weight (g) loss provoked by CIS in male (**A**,**C**) and female (**B**,**D**) mice treated with DULO and HRW alone and in combination for 30 consecutive days. In all tests, * indicates significant differences vs. corresponding mice treated with VEH-VEH-VEH, VEH-DULO-VEH, or VEH-VEH-HRW; @, CIS-VEH-VEH; and #, CIS-VEH-HRW (*p* < 0.05, one-way ANOVA plus Holm–Šídák multiple comparisons test). Mean values ± SEM; n = 8 animals.

The coadministration of DULO with HRW also avoided the grip strength loss provoked by CIS from days 13 to 30 of treatment in male (*p* < 0.001; one-way ANOVA; Figure 5A) and female mice (*p* < 0.001; one-way ANOVA; Figure 5B). As with allodynia, DULO had stronger inhibitory effects than HRW, in this case from days 25 to 30 of treatment, in both male and female mice. Additionally, DULO given alone had comparable effects to the combination between days 2–23 and 30 of treatment in male mice (*p* < 0.001; one-way ANOVA; Figure 5A) and from days 2 to 30 of treatment in female mice (*p* < 0.001; one-way ANOVA; Figure 5B). The administration of HRW also prevented the loss of grip strength from days 2 to 18 in male animals and from days 2 to 23 of treatment in female mice, but its inhibitory actions decreased as compared with those produced by DULO plus HRW from days 20 to 30 of treatment in male mice and from days 23 to 30 in female mice. The AUC analysis revealed the complete inhibition of CIS-induced grip strength loss in male (Figure 6A) and female (Figure 6B) mice treated with DULO and HRW combined and the partial inhibition induced by each treatment alone (*p* < 0.001; one-way ANOVA) (Table S1).

Regarding the body weight loss observed in CIS-injected male (Figure 5C) and female (Figure 5D) mice, preventive treatment with DULO and HRW alone or combined did not reverse it completely in any of the cases. Therefore, the effects of the three treatments in CIS-injected mice were lower than those on the corresponding VEH-injected animals from days 20 to 30 in male (*p* < 0.001; one-way ANOVA; Figure 5C) and female mice (*p* < 0.001; one-way ANOVA; Figure 5D). The AUC analysis also revealed that there were no significant differences between the effects of treatments on the body weight loss caused by CIS in male (Figure 6C) and female mice (Figure 6D; Table S1).

In all tests, administration of DULO or HRW, alone or in combination, did not induce significant changes in male or female animals injected with VEH. Furthermore, no significant differences were detected between treatments in either male or female mice injected with VEH or CIS.

### 3.3. Treatment with DULO and HRW Alone and Combined Avoided the Development of Emotional Disorders Associated with CIS-Induced Chemotherapy

**Figure 7:**
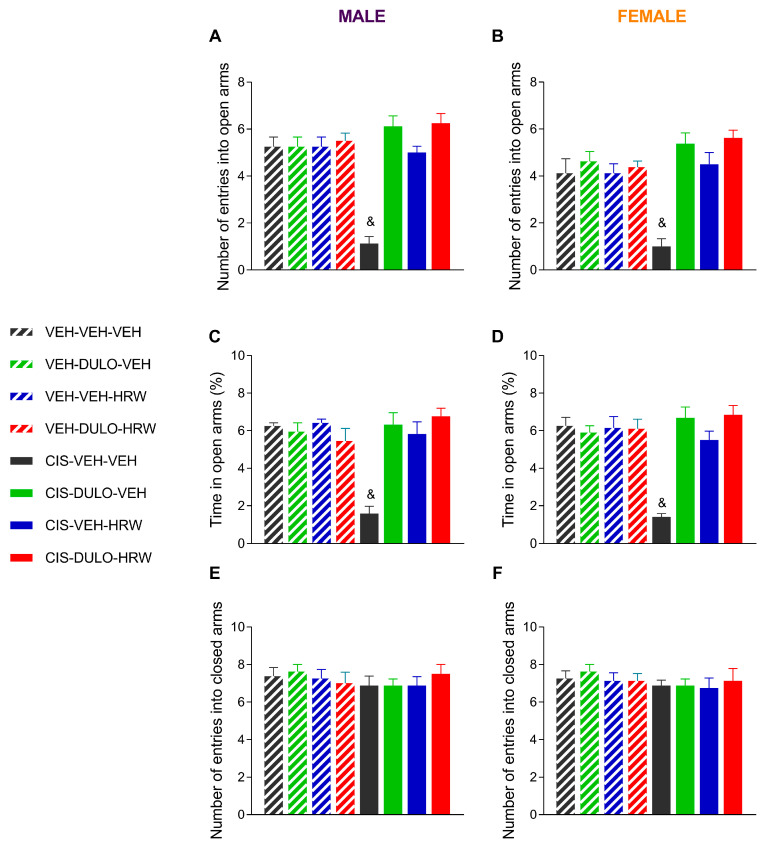
Treatment with DULO, HRW, and DULO plus HRW inhibited the anxiety-like behaviors provoked by CIS in male and female mice. The effects of the prophylactic administration of DULO, HRW, and DULO plus HRW on the number of entrances into the open arms (**A**,**B**), the percentage of time spent in them (**C**,**D**), and the number of entrances into the closed arms (**E**,**F**) for male and female mice injected with VEH or CIS in the EPM test are shown. In all graphs, & indicates significant differences vs. the rest of the groups (*p* < 0.05, one-way ANOVA plus Holm–Šídák multiple comparisons test). Mean values ± SEM; n = 8 animals.

**Figure 8:**
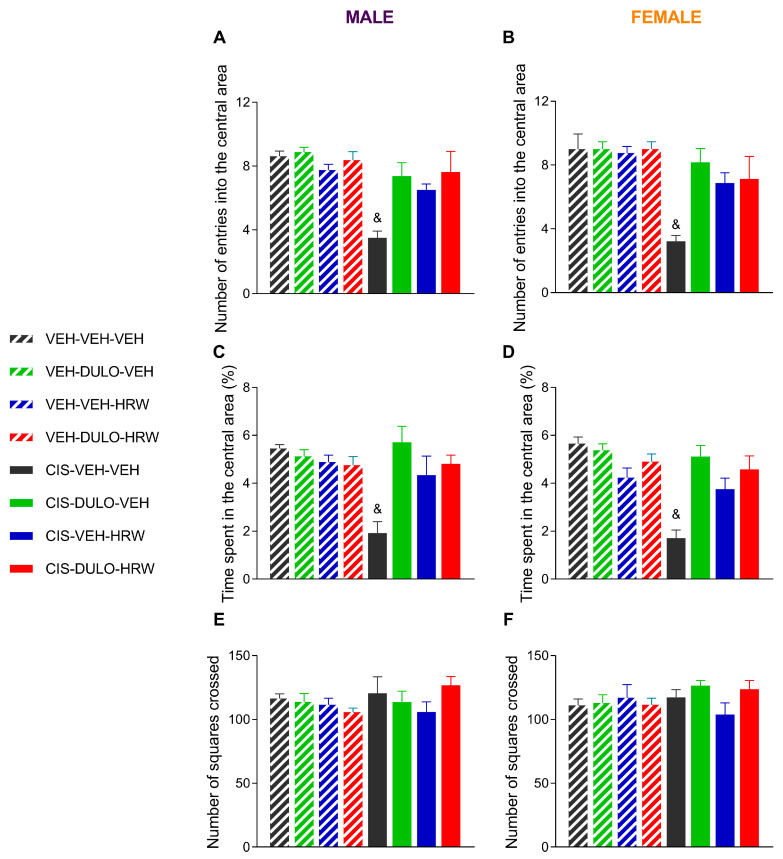
Treatment with DULO, HRW, and DULO plus HRW inhibited the anxiety-like behaviors provoked by CIS in male and female mice. The effects of the prophylactic administration of DULO, HRW, and DULO plus HRW on the number of entrances into (**A**,**B**) and the percentage of time spent in the central area (**C**,**D**), as well as the number of squares crossed (**E**,**F**), by male and female mice injected with VEH or CIS in the OF test are displayed. In all graphs, & indicates significant differences vs. the rest of the groups (*p* < 0.05, one-way ANOVA plus Holm–Šídák multiple comparisons test). Mean values ± SEM; n = 8 animals.

Our results confirmed the anxious-like behaviors (Figure 7 and Figure 8) that accompany CIS-induced neuropathy and that they equally manifested in both sexes. Indeed, three-way ANOVAs revealed significant effects of injection, treatment, and their interaction on the number of entries into (Figure 7A,B) and the time expended in the open arms of the EPM test (Figure 7C,D) for male and female mice (Table 4). For both sexes, the diminished number of entrances and the short time spent to the open arms suggested anxious-like behavior in CIS-injected animals that was reversed by prophylactic treatment with DULO, HRW, and DULO plus HRW during 30 consecutive days (*p* < 0.001; one-way ANOVA). No significant differences in the quantity of entrances into the closed arms (Figure 7E,F) were detected among any of the groups.

We further assessed the effects of DULO, HRW, and DULO plus HRW via the OF test to confirm the anxiolytic effects of these treatments in the EPM test. As above, the three-way ANOVAs revealed significant effects of injection, treatment, and their interaction for the number of entries into the central area (Figure 8A,B) and the time expended in the central area (Figure 8C,D) for male and female mice (Table 5). The reduced number of entries into the central area and the brief time spent in it confirmed the anxious-like behavior observed in CIS-injected mice, which was reversed, as above, by prophylactic treatment with DULO, HRW, and DULO plus HRW during 30 consecutive days (*p* < 0.001; one-way ANOVA). No significant differences in the quantity of squares crosses were identified among the groups (Figure 8E,F).

In both tests and sexes, no effects of treatments were detected in VEH-injected animals, nor were there differences between the effects produced by each treatment in these animals.

**Figure 9:**
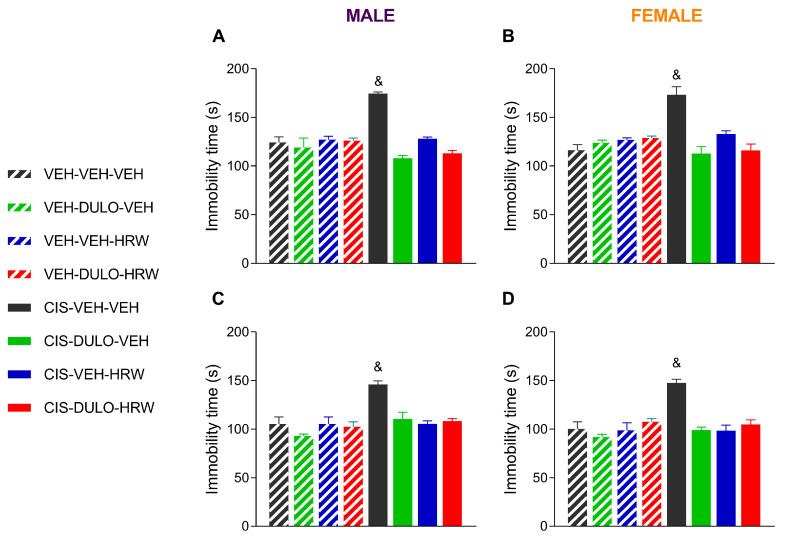
Treatment with DULO, HRW, and DULO plus HRW inhibited the depressive-like behaviors provoked by CIS in male and female mice. The effects of the prophylactic administration of DULO, HRW, and DULO plus HRW on the time that male and female mice injected with VEH or CIS remained immobile (s) in the TST (**A**,**B**) and FST (**C**,**D**) are displayed. In all graphs, & indicates significant differences vs. the rest of the groups (*p* < 0.05, one-way ANOVA plus Holm–Šídák multiple comparisons test). Mean values ± SEM; n = 8 animals.

Our data likewise verified an increase in the time that male and female CIS-injected animals remained immobile in the TST (Figure 9A,B) and the FST (Figure 9C,D) (*p* < 0.001; one-way ANOVA compared with corresponding VEH-VEH-VEH-treated subjects), thus confirming a depressive-like status that was fully prevented by prophylactic treatment with DULO, HRW, or DULO plus HRW during 30 consecutive days (*p* < 0.001; one-way ANOVA). Indeed, the three-way ANOVAs revealed significant effects of injection, treatment, and their interaction in both tests (Table 6).

In all tests, treatment with DULO and HRW alone and combined did not produce any effect on VEH-injected male or female animals. Additionally, the effects of each treatment in VEH-injected animals were not significantly different between groups.

### 3.4. The Impact of Treatment with DULO and HRW Alone and Combined on the Oxidative Stress, Inflammatory Responses, and Plasticity Changes Triggered by CIS in the DRG and AMG of Male and Female Mice

**Figure 10:**
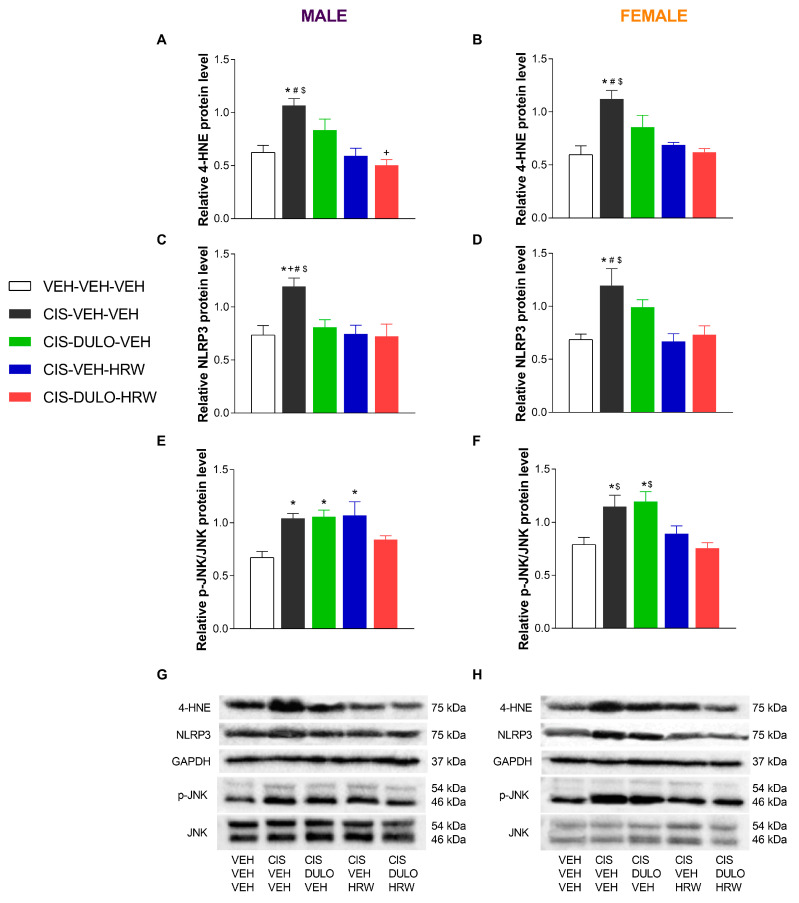
The effects of treatment with DULO and HRW alone and combined on the oxidative, inflammatory, and plasticity changes triggered by CIS in the DRG of male and female mice. The relative protein levels of 4-HNE (**A**,**B**), NLRP3 (**C**,**D**), and p-JNK (**E**,**F**) in the DRG of male and female CIS-injected mice are shown. Control animals treated with VEH-VEH-VEH are also shown, as are representative blots of 4-HNE, NLRP3, and p-JNK/JNK from the DRG of male (**G**) and female (**H**) mice are also shown. In all panels, * indicates significant differences vs. corresponding mice treated with VEH-VEH-VEH; +, CIS-DULO-VEH; #, CIS-VEH-HRW; and $, CIS-DULO-HRW (*p* < 0.05, one-way ANOVA plus Holm–Šídák multiple comparisons test). Mean values ± SEM; n = 4–5 samples per group.

**Figure 11:**
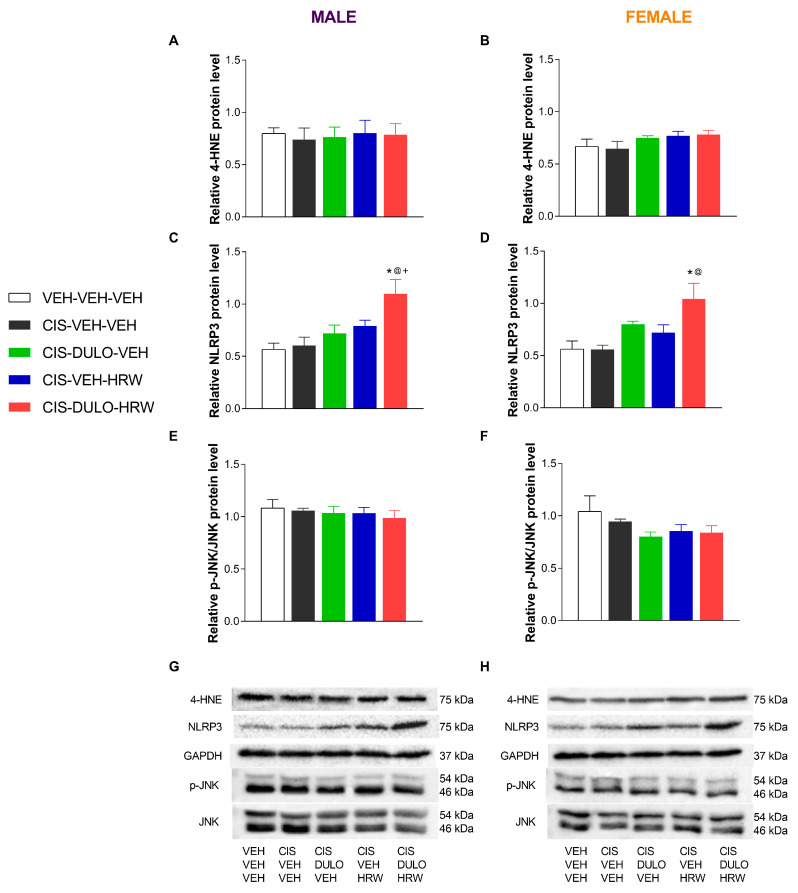
The effects of treatment with DULO and HRW alone and combined on the oxidative, inflammatory, and plasticity changes in the AMG of male and female mice. The relative protein levels of 4-HNE (**A**,**B**), NLRP3 (**C**,**D**), and p-JNK (**E**,**F**) in the AMG of male and female CIS-injected mice are shown. Control animals treated with VEH-VEH-VEH are also shown, as are representative blots of 4-HNE, NLRP3, and p-JNK/JNK from the AMG of male (**G**) and female (**H**) mice. In all panels, * indicates significant differences vs. corresponding mice treated with VEH-VEH-VEH; +, CIS-DULO-VEH; and @, CIS-VEH-VEH (*p* < 0.05, one-way ANOVA plus Holm–Šídák multiple comparisons test). Mean values ± SEM; n = 4–5 samples per group.

Because of the well-known participation of oxidative stress, inflammation, and neuronal plasticity in the progression of neuropathy caused by CIS and the accompanying mental diseases, we evaluated the impact of treatment with DULO and HRW alone and combined on the protein levels of 4-HNE, the NLRP3 inflammasome, and p-JNK in the DRG and AMG of CIS-injected male and female mice (Figure 10 and Figure 11).

Two-way ANOVAs revealed significant effects of treatment on the expression of 4-HNE, NLRP3, and p-JNK in the DRG and the NLRP3 in the AMG (Table 7). Indeed, the peripheral oxidative and inflammatory reactions triggered by CIS were proved with the increased expression of 4-HNE (*p* < 0.001, one-way ANOVA; Figure 10A,B) and NLRP3 (*p* < 0.002, one-way ANOVA; Figure 10C,D) in the DRG of male and female mice. Additionally, whereas the administration of DULO and HRW alone and combined normalized the oxidative and inflammatory reactions caused by CIS in the DRG of both male and female mice, an overexpression of NLRP3 was identified in the AMG of male and female mice treated with DULO plus HRW (*p* < 0.003, one-way ANOVA; Figure 11C,D). CIS injection further induced JNK phosphorylation in the DRG of male (*p* < 0.002, one-way ANOVA; Figure 10E) and female mice (*p* < 0.001, one-way ANOVA; Figure 10F). Nonetheless, only combined treatment with DULO and HRW reversed the JNK phosphorylation in the DRG of CIS-injected male mice, whereas HRW alone and combined with DULO normalized the JNK activation in the DRG of CIS-injected female mice.

Nonsignificant changes in the expression of 4-HNE (Figure 11A,B) and p-JNK (Figure 11E,F) were detected in the AMG of male and female mice.

We also analyzed the effectiveness of treatment with DULO and HRW alone and combined on the protein levels of the antioxidant enzymes HO-1, SOD-1, and NQO1 in the DRG and AMG of male and female mice. The two-way ANOVAs revealed significant effects of treatment only on the expression of HO-1 and SOD-1 in the DRG (Table 8).

**Figure 12:**
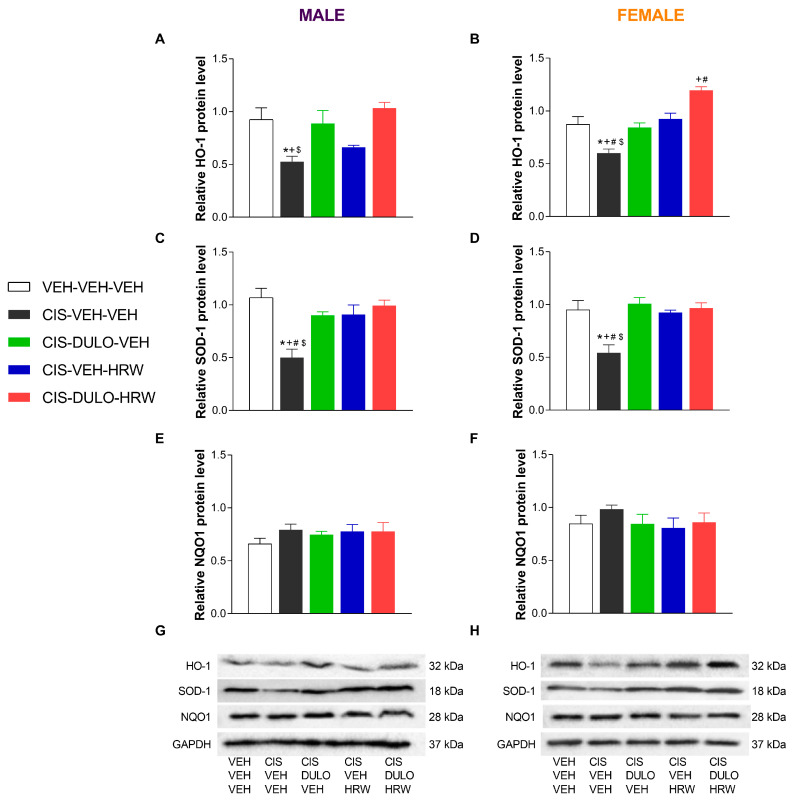
The impact of administering DULO and HRW separately and in combination on the levels of antioxidant enzymes in the DRG of CIS-injected male and female mice. The relative protein levels of HO-1 (**A**,**B**), SOD-1 (**C**,**D**), and NQO1 (**E**,**F**) in the DRG of male and female CIS-injected mice are shown. Control animals treated with VEH-VEH-VEH are also shown, as are representative blots of HO-1, SOD-1, and NQO1 from the DRG of male (**G**) and female (**H**) mice. In all panels, * indicates significant differences vs. corresponding mice treated with VEH-VEH-VEH; +, CIS-DULO-VEH; #, CIS-VEH-HRW; and $, CIS-DULO-HRW (*p* < 0.05, one-way ANOVA plus Holm–Šídák multiple comparisons test). Mean values ± SEM; n = 4–5 samples per group.

Our data confirmed the diminished expression of HO-1 in the DRG of CIS-injected male (*p* < 0.001, one-way ANOVA; Figure 12A) and female mice (*p* < 0.001, one-way ANOVA; Figure 12B) as compared with their corresponding VEH-VEH-VEH treated animals. In the same line, CIS also diminished the levels of SOD-1 in the DRG of male (*p* < 0.001, one-way ANOVA; Figure 12C) and female mice (*p* < 0.001, one-way ANOVA; Figure 12D). Results also showed that treatment with DULO and DULO plus HRW, but not with HRW alone, normalized the downregulation of HO-1 in the DRG of male mice, while all treatments inhibited it in female mice. In this sex, the enhanced expression of HO-1 induced by DULO plus HRW was higher than that produced by DULO and HRW given separately (Figure 12B). In both sexes, the downregulation of SOD-1 in the DRG was normalized by DULO and HRW administered alone and combined (Figure 12C,D). Nonchanges in the expression of NQO1 were identified in the DRG of male and female CIS-injected mice (Figure 12E,F).

**Figure 13:**
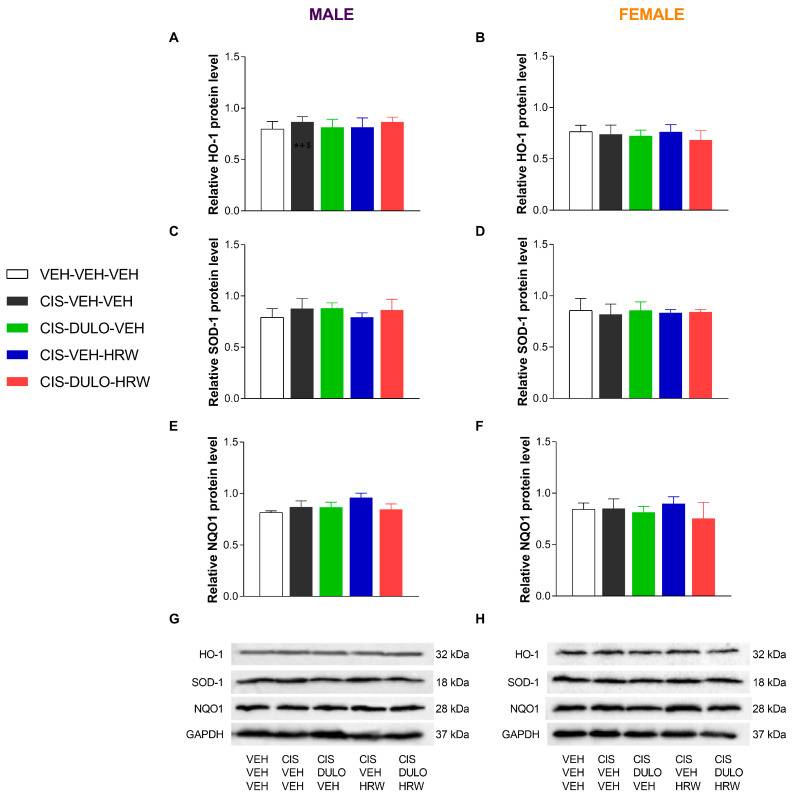
The impact of administering DULO and HRW separately and in combination on the levels of antioxidant enzymes in the AMG of CIS-injected male and female mice. The relative protein levels of HO-1 (**A**,**B**), SOD-1 (**C**,**D**), and NQO1 (**E**,**F**) in the AMG of male and female CIS-injected mice are shown. Control animals treated with VEH-VEH-VEH are also shown, as are representative blots of HO-1, SOD-1, and NQO1 from the AMG of male (**G**) and female (**H**) mice. Mean values ± SEM; n = 4–5 samples per group.

Regarding the AMG, nonchanges in the expression of HO-1, SOD-1, and NQO1 were detected in male (Figure 13A,C,E) and female mice (Figure 13B,D,F).

## 4. Discussion

This study showed, for the first time, that cotreatment with DULO and HRW exhibited efficacious inhibitory effects in CIS-induced neuropathy and muscle weakness, and that these effects were higher than those produced by each treatment administered alone. This cotreatment also avoided the emotional disorders associated with neuropathy but only partially reversed the weight loss caused by CIS. Most of these effects were mainly accomplished through stabilizing the inflammatory, oxidative, and plasticity reactions provoked by CIS in the DRG. Therefore, our results reveal that DULO combined with HRW might be a safe way to treat CIS-induced neuropathy and the physical and emotional deficiencies that come with it. This combined treatment would greatly improve the quality of life for people receiving this type of chemotherapy.

As reported in other studies, our results confirmed similar responses between sexes regarding CIS-induced mechanical allodynia, in contrast to the different progression of cold allodynia, being more pronounced in females [30,31,32]. We also demonstrated that the combined administration of low doses of DULO with HRW avoided the development of mechanical allodynia in both sexes, which was only partially inhibited with the administration of either treatment given alone. Our results are in accordance with the modest antiallodynic actions produced by low doses of DULO in CIS-, paclitaxel- and vincristine-induced neuropathic pain in rodents [1,8,9,33]. These findings are also consistent with the moderate recommendation made by the ASCO guideline for the use of DULO in the treatment of chemotherapy-induced peripheral neuropathy [34]. The mechanical antiallodynic effects of HRW in CIS-injected mice have been previously demonstrated [23], showing that the administration of HRW at 0.30 mM during 30 consecutive days prevented the development of mechanical allodynia. This study further revealed that administration of HRW at 0.15 mM produced only limited inhibition of the mechanical allodynia caused by this chemotherapeutic agent. Interestingly, the fact that combined treatment with low doses of DULO and HRW avoided the mechanical allodynia caused by CIS in both sexes revealed that this combination might be an interesting approach for treating mechanical allodynia.

Furthermore, our findings also indicated that the progression of cold allodynia induced by CIS differed between sexes, with female mice exhibiting more pronounced effects. This finding might be explained, at least in part, by the higher sensitivity to cold stimulus observed in CIS-injected females than in males. These different pain perceptions between sexes may be in part attributed to their divergent hormonal profiles, where elevated testosterone levels in males augment the pain threshold and fluctuations in estrogen levels in females heighten pain intensity and perception [35]. The results also showed that DULO combined with HRW stopped CIS-induced cold allodynia completely in male mice but only partially in females. Moreover, the thermal antiallodynic actions of DULO combined with HRW were greater that those of DULO and HRW separately in males and those of HRW in female mice. Other authors have also shown that DULO reduced cold allodynia provoked by oxaliplatin in male mice and that caused by osteoarthritis in male and female animals [7,33,36] and that HRW mitigated nerve-injury-induced cold allodynia [11]. In addition, the full prevention of CIS-caused cold allodynia in males, but only partial prevention in female mice, revealed that sex affects the outcomes of this combined therapy. Accordingly, several studies have demonstrated that sex also influenced the effectivity of some treatments, with treatments being more effective in male than female animals [35]. Nonetheless, the increased cold antiallodynic effects of DULO plus HRW vs. those of HRW and DULO given alone corroborated the greater effectiveness of this combination. Accordingly, other studies have also displayed enhanced effects of DULO combined with minocycline in inhibiting the mechanical allodynia caused by oxaliplatin [7]. Last, considering the absence of adverse effects produced by HRW, its combination with DULO has the potential to be clinically applied in the future.

Cachexia and muscle atrophy are frequent adverse effects in cancer patients receiving chemotherapy. They make patients’ quality of life worse and chemotherapy less effective, and they may even shorten patients’ lives [37,38]. Therefore, it is important to identify approaches to prevent and/or attenuate muscle and weight loss development throughout CIS therapy in humans. These adverse reactions have also been demonstrated in preclinical animal models [39,40]. Our results confirmed these outcomes by showing that CIS injection resulted in grip strength deficits and body weight loss in mice of both sexes and that these deficits were more pronounced in female mice [41,42,43], endorsing the influence of sex in this negative effect of CIS in mice. It is noteworthy that combined administration of DULO and HRW prevented the grip strength loss stimulated by CIS in both sexes and that this effect was more marked than that produced by each treatment alone in male mice and by HRW in female mice during the last phases of the therapy. In both sexes, DULO exhibited greater inhibitory actions than HRW after 25 to 30 days of treatment. These findings were in part due to the low dose of HRW used in this study, which did not entirely reverse the loss of grip strength caused by CIS as it did when given at 0.3 mM [23]. Even so, the mixture of DULO and HRW improved the inhibitory actions of these treatments, which could represent an important benefice in the quality of life of patients by preventing muscle atrophy.

Predictably, our data confirmed that the body weight loss provoked by CIS was greater in female than in male mice. Nevertheless, and besides these differences between sexes, prophylactic treatment with DULO and HRW, given alone or combined, could not completely prevent the weight loss provoked by CIS in male or female mice. These results confirmed the low effectivity of DULO in stopping weight loss in some preclinical models [1]. Nevertheless, the inhibitory effects of both treatments alone and combined seemed to be higher in female mice. This was corroborated with significant differences in body weight observed between CIS-injected female animals treated with VEH vs. those receiving DULO, HRW, and DULO plus HRW from days 16 to 30 of treatment. Meanwhile, significant differences between CIS-injected male mice receiving VEH and those cotreated with DULO plus HRW were detected only at days 27 and 30 of treatment.

In summary, the combined treatment prevented the deficit of muscle strength but only partially protected against the body weight loss triggered by CIS in male and female mice. These dissimilar effects produced by the combined treatment might be related to the fact that while the body weight is regulated by the central nervous system, grip strength of the hind extremities of the animals is mainly regulated by the peripheral nervous system. Thus, the normalization of the oxidative stress, inflammatory, and/or plasticity changes produced by the combined treatment in the DRG of CIS-injected animals might be responsible for the inhibition of muscular deficits induced by the cotreatment. Other studies have indicated that other treatments, for instance capsaicin, can alleviate and ameliorate CIS-induced body weight and muscle strength loss in mice [44].

People who receive CIS as a chemotherapy treatment commonly develop signs of anxiety and depression [45], which make it harder for them to stick with their cancer treatment, seriously affecting their quality of life. Sometimes, these anxious and depressive conditions compel the patient to take anxiolytic and/or antidepressant drugs with well-documented side effects, such as cardiovascular adverse reactions, gastrointestinal issues, disturbed sleep, apathy, and fatigue [46]. As a result, it is crucial to search for a therapy that not only prevents the development of neuropathy but avoids the emotional disorders associated with it to prevent the premature discontinuation of chemotherapy. In conformity with other preclinical studies, this research proved that chemotherapy induced by CIS was associated with anxiogenic and depressive-like behaviors in both male and female mice [47]. As a result, the number of entries and the time that mice spent into the open arms of the EPM, as well as the number of entries and the time spent in the central area of the OF test, were reduced in CIS-injected animals treated with VEH. Conversely, the time that mice remained stationary in the TST and FST was increased in both sexes. All of these cases confirmed that the sex of the animals did not influence these behaviors. Furthermore, the lack of CIS and treatment effects on the animals’ locomotor activity was evidenced by the absence of alterations in the number of entries into the closed arms in the EPM test and the number of squares traversed in the OF test.

Moreover, individual and combined treatment with DULO and HRW prevented the anxiodepressive-like conditions linked to CIS-induced peripheral neuropathy in male and female mice. It is interesting to note that while both treatments given separately presented unsatisfactory analgesia and muscular deficit recuperation, as neither could completely prevent the development of allodynia and/or grip strength deficits caused by CIS, each of them avoided the development of affective disorders accompanying CIS-induced chemotherapy. These outcomes suggested that the full reversion of pain was not required to avoid the derivate emotional diseases. Nonetheless, as expected, combined treatment with DULO plus HRW also prevented the anxiety- and depressive-like behaviors accompanying neuropathy provoked by CIS in male and female mice. These findings agreed with the anxiolytic and antidepressant activities produced by HRW in animals with inflammatory and neuropathic pain instigated by a nerve injury [11,22]. More interesting is the detail that in contrast to the recognized side effects induced by high doses of DULO [5], no adverse effects have been described for HRW [48]. Therefore, considering that we used a low dose of DULO (5 mg/kg), its combination with HRW might be considered as an alternative therapy for preventing the nociceptive and affective symptoms related to CIS-induced chemotherapy.

Oxidative stress, inflammation, and changes in neuronal plasticity are the primary causes of neuropathic pain induced by CIS [19,40,49,50]. The excessive ROS production occasioned by this antitumoral drug results in an unsatisfactory synthesis of antioxidant proteins, thus creating a disequilibrium among the oxidant and antioxidant systems of the organism [49]. Accordingly, increased expression of 4-HNE was demonstrated in the DRG of male and female CIS-injected mice, thus confirming the oxidative stress caused by this antineoplastic drug in the peripheral nervous system. These findings were substantiated by the reduced expression of the antioxidant enzymes HO-1 and SOD-1 in this tissue. Concomitantly, enhanced levels of 4-HNE and decreased expression of HO-1 in the PFC, as well as reduced levels of SOD-1, in the DRG, PFC, and hippocampus of CIS-injected animals have also been demonstrated [18,51,52]. In contrast to these results, nonchanges in the expression of 4-HNE, HO-1, SOD-1, and NQO1 in the AMG of CIS-injected mice were detected, thus suggesting that this brain area seems not to be severely affected by this type of chemotherapy as it is with paclitaxel, which increases the AMG expression of 4-HNE [53]. Nevertheless, because the central nucleus of the AMG is involved in CIS-induced body weight loss by regulating glutamate receptor signaling [54], it is possible that some oxidative responses may take place in specific nuclei of the AMG. More experiments are needed to demonstrate this hypothesis.

Interestingly, the administration of DULO and HRW, alone and combined, normalized the upregulation of 4-HNE in the DRG of male and female animals, with the effects of both drugs administered together being greater than those of DULO alone in male mice. Our data also displayed that treatment with DULO alone and combined with HRW normalized the downregulation of HO-1 in the DRG of male mice, while all treatments regularized the HO-1 expression in female mice. In this case, the upregulation of HO-1 induced by DULO plus HRW was higher than those produced by each of them given separately. The results indicate that sex-specific differences may play a role in the regulation of HRW-induced HO-1 expression in the DRG of CIS-injected mice. It appears that HRW treatment requires the presence of estrogen and progesterone to restore the diminished expression of HO-1 induced by CIS in the DRG. Moreover, the increased expression of HO-1 resulting from the combined treatment in female mice may be attributed to the modulation of this antioxidant enzyme induced by the administration of HRW alone. In addition, considering that several antioxidant agents, such as trimetazidine and phytic acid, reduce the nociceptive responses caused by CIS [55,56], the antioxidant actions of our treatments might explicate, at least in part, the inhibition of the allodynia and grip strength deficits in animals treated with DULO and HRW administered alone, as well as the greater actions produced by its coadministration, in male and female animals. The last point was reinforced by the fact that the combined treatment showed greater inhibition of 4-HNE than DULO alone in male mice, as well as by the increased HO-1 caused by the combined treatment in female mice compared with that caused by DULO and HRW separately. Furthermore, the normalization of CIS-induced SOD-1 downregulation produced by both treatments, given alone and combined, in both sexes also suggests the participation of this antioxidant enzyme in the antiallodynic actions and the recuperation of muscle loss produced by these treatments. In agreement with us, previous studies have exhibited the reversal of the analgesic effects of HRW with the coadministration of specific inhibitors of the Nrf2/HO-1 signaling pathway in animals with paclitaxel-induced neuropathy [53]. Finally, the nonchanges in the expression of NQO1 in the DRG of CIS-injected mice treated with VEH, DULO, HRW, and DULO plus HRW were consistent with those observed in paclitaxel-injected and nerve-injured mice treated with HRW [11,53], suggesting that this detoxicant enzyme is not involved in these activities.

NLRP3 is the most extensively studied inflammasome. A growing body of research has revealed that the activation of this inflammasome is involved in the development of chronic pain generated by osteoarthritis, peripheral inflammation, nerve injury, and chemotherapy [57]. The pharmacological inhibition of NLRP3 inflammasome activation showed therapeutic effects in the above-mentioned conditions, revealing that its blockage is essential for treating chronic pain [58,59]. Furthermore, this inflammasome is linked with multiple mental illnesses, for instance, anxiety and depression associated with chronic pain caused by spinal cord injuries, persistent inflammation, or chemotherapy [60,61,62]. As expected, increased levels of the inflammasome NLRP3 in the DRG of CIS-injected male and female mice were confirmed in our study. Moreover, the prophylactic administration of DULO and HRW alone and combined regularized the overexpression of this inflammasome. The anti-inflammatory effects of HRW here confirmed those previously demonstrated in paclitaxel-injected mice. It was further revealed that DULO combined with HRW also reduced the inflammatory reactions provoked by CIS in male and female mice. The anti-inflammatory properties of DULO have also been described in BV-2 microglial cells by diminishing the upregulation of nitric oxide and the phosphorylation of IκBα and Akt provoked by LPS [63]. Our data further demonstrated the capacity of this drug to block NLRP3 activation in the DRG of CIS-injected mice, which might also contribute to its antinociceptive activity. The expression of NLRP3 was not altered by CIS in the AMG as occurred in paclitaxel-injected mice [53], thus showing the dissimilar expression of this inflammasome in this brain area according to the type of antineoplastic drug. Unpredictably, the coadministration of DULO plus HRW increased the expression of NLRP3 in the AMG of CIS-injected male and female mice, showing the opposed effects of this combined treatment in the peripheral and central nervous system of these animals. A plausible explanation for the unexpected upregulation of NLRP3 induced by the administration of DULO plus HRW in the AMG of CIS-injected mice may involve several factors: (i) the expression of this inflammasome was evaluated in the whole AMG, but it is noteworthy that anxiety- and depressive-like behaviors are predominantly regulated by the basolateral AMG, making it possible that increased expression of NLRP3 and its reversion with the combined treatment may occur specifically in this region; (ii) the fact that the increased expression of NLRP3 demonstrated in the PFC of CIS-injected animals was reversed by the administration of HRW [23] suggests that the anxiolytic and antidepressant actions elicited by DULO and HRW may also take place in this brain area by modulating NLRP3 upregulation; (iii) a potential compensatory mechanism between the AMG and PFC in regulating NLRP3 expression could also be involved in the modulation of the emotional disorders caused by this cotreatment in CIS-injected mice. Nevertheless, further experiments are required to validate these hypotheses.

The involvement of MAPK signaling pathways in the development and progression of CIS-induced neuropathic pain has also been demonstrated; thus, elevated levels of p-JNK have been detected in the DRG of these animals [19]. Our results supported these findings in both male and female mice. They also showed that DULO and HRW given alone did not reverse the higher levels of p-JNK in the DRG of male mice, but they inhibited it when given together. Similar results were obtained in the DRG of female mice, but in this case, both HRW alone and combined with DULO inhibited the upregulation of p-JNK. Other authors have also reported that molecular hydrogen exhibits its anti-inflammatory and antioxidative properties through inhibiting the JNK pathway [64]. That is, this gas alleviates postoperative pain and mitigates the progression of osteoarthritis by suppressing inflammation through inhibiting JNK activation [64,65]. Thus, the prevention of p-JNK activation induced by DULO mixed with HRW in the DRG might also be involved in the improvement of the inhibitory actions induced by this cotreatment in CIS-injected male and female mice.

A limitation of this study is the absence of changes induced by CIS and/or the treatments (DULO and HRW, both individually and in combination) in the expression of the majority of proteins analyzed in the AMG, which did not permit the elucidation of the primary mechanisms underlying the efficacy of these treatments in mitigating the anxiety- and depressive-like behaviors induced by CIS in both male and female mice. The heightened expression of NLRP3 resulting from cotreatment with DULO and HRW did not corroborate our behavioral findings, indicating that additional factors may contribute to the modulation of these emotional disorders linked to this chemotherapy regimen. Furthermore, quantifying systemic or tissue hydrogen levels to verify HRW delivery and duration, along with demonstrating that HRW and/or DULO do not diminish CIS cytotoxicity via in vitro tumor cell viability assays, would substantiate the efficacy of the combined treatment in alleviating CIS-induced neuropathy and strengthen the translational relevance of these results.

## 5. Conclusions

This study proved that prophylactic treatment with DULO combined with HRW prevented the mechanical allodynia and muscle strength deficits caused by CIS in mice of both sexes. The combined treatment also prevented cold allodynia in male mice but only reduced it in females. Moreover, the weight loss caused by CIS in both sexes was not completely counteracted by the combined therapy, although all treatments prevented the anxiodepressive-like behaviors accompanying CIS-induced neuropathy. The antiallodynic effects and prevention of muscular deficits caused by this combination may primarily be attributed to the normalization of inflammatory, oxidative, and synaptic plasticity responses provoked by CIS in the DRG of these animals. These findings suggest that DULO combined with HRW could be an effective and safe way to treat CIS-induced neuropathy and its associated emotional disorders, which would greatly improve the quality of life of people undergoing this type of chemotherapy.

## Figures and Tables

**Table 1:** Primary antibodies employed in this investigation.

Antibody	Molecular Weight (kDa)	Dilution	Commercial Supply
4-HNE	75	1:100	Abcam, Cambridge, UK
NLRP3	75	1:200	Adipogen Life Sciences, Epalinges, Switzerland
p-JNK	54/46	1:250	Cell Signaling Technology, Danvers, MA, USA
JNK	54/46	1:250	Cell Signaling Technology, Danvers, MA, USA
HO-1	32	1:200	Abclonal Technology, Woburn, MA, USA
SOD-1	18	1:150	Novus Biologic, Littleton, CO, USA
NQO1	28	1:250	Merck, Billerica, MA, USA
GAPDH	37	1:5000	Merck, Billerica, MA, USA

**Table 2:** Summary of three-way repeated measure ANOVAs performed with data on mechanical allodynia, thermal allodynia, grip strength, and body weight obtained at different times (0, 2, 4, 6, 9, 11, 13, 16, 18, 20, 23, 25, 27 and 30 days) after VEH or CIS injection in male and female mice.

	Mechanical Allodynia	Thermal Allodynia	Grip Strength	Body Weight
Injection	*p* < 0.001	*p* < 0.001	*p* < 0.001	*p* < 0.009
Sex	*p* < 0.328	*p* < 0.048	*p* < 0.028	*p* < 0.002
Time	*p* < 0.001	*p* < 0.001	*p* < 0.001	*p* < 0.001
Injection × Sex	*p* < 0.729	*p* < 0.080	*p* < 0.321	*p* < 0.767
Injection × Time	*p* < 0.001	*p* < 0.001	*p* < 0.001	*p* < 0.001
Sex × Time	*p* < 0.988	*p* < 0.945	*p* < 0.126	*p* < 0.001
Injection × Sex × Time	*p* < 0.968	*p* < 0.864	*p* < 0.761	*p* < 0.001

**Table 3:** Summary of three-way repeated measures ANOVAs performed with data on mechanical allodynia, thermal allodynia, grip strength, and body weight obtained at different times (0, 2, 4, 6, 9, 11, 13, 16, 18, 20, 23, 25, 27 and 30 days) after VEH or CIS injection in male and female mice treated with VEH, DULO, HRW, or DULO plus HRW.

	Mechanical Allodynia	Thermal Allodynia	Grip Strength	Body Weight
Treatment	*p* < 0.001	*p* < 0.001	*p* < 0.001	*p* < 0.001
Sex	*p* < 0.122	*p* < 0.015	*p* < 0.002	*p* < 0.001
Time	*p* < 0.001	*p* < 0.001	*p* < 0.001	*p* < 0.001
Treatment × Sex	*p* < 0.813	*p* < 0.016	*p* < 0.848	*p* < 0.353
Treatment × Time	*p* < 0.001	*p* < 0.001	*p* < 0.001	*p* < 0.001
Sex × Time	*p* < 0.984	*p* < 0.410	*p* < 0.330	*p* < 0.001
Treatment × Sex × Time	*p* < 0.999	*p* < 0.999	*p* < 0.921	*p* < 0.001

**Table 4:** Summary of three-way ANOVAs performed with data on EPM test obtained at 30 days after VEH or CIS injection from male and female mice treated with VEH, DULO, HRW, or DULO plus HRW.

	Number of Entries into Open Arms	Time in Open Arms	Number of Entries into Closed Arms
Injection	*p* < 0.032	*p* < 0.001	*p* < 0.158
Sex	*p* < 0.523	*p* < 0.884	*p* < 0.736
Treatment	*p* < 0.001	*p* < 0.001	*p* < 0.879
Injection × Sex	*p* < 0.216	*p* < 0.830	*p* < 0.840
Injection × Treatment	*p* < 0.001	*p* < 0.001	*p* < 0.484
Sex × Treatment	*p* < 0.986	*p* < 0.781	*p* < 0.997
Injection × Sex × Treatment	*p* < 0.867	*p* < 0.905	*p* < 0.965

**Table 5:** Summary of three-way ANOVAs performed with data from OF test obtained at 30 days after VEH or CIS injection with male and female mice treated with VEH, DULO, HRW, or DULO plus HRW.

	Number Entries into Central Area	Time in Central Area	Number Squares Crossed
Injection	*p* < 0.001	*p* < 0.001	*p* < 0.188
Sex	*p* < 0.384	*p* < 0.338	*p* < 0.740
Treatment	*p* < 0.001	*p* < 0.001	*p* < 0.397
Injection × Sex	*p* < 0.548	*p* < 0.368	*p* < 0.982
Injection × Treatment	*p* < 0.001	*p* < 0.001	*p* < 0.182
Sex × Treatment	*p* < 0.902	*p* < 0.748	*p* < 0.796
Injection × Sex × Treatment	*p* < 0.846	*p* < 0.910	*p* < 0.671

**Table 6:** Summary of three-way ANOVAs performed with data from TST and FST obtained at 30 days after VEH or CIS injection from male and female mice treated with VEH, DULO, HRW, or DULO plus HRW.

	Immobility Time TST	Immobility Time FST
Injection	*p* < 0.001	*p* < 0.001
Sex	*p* < 0.581	*p* < 0.172
Treatment	*p* < 0.001	*p* < 0.001
Injection × Sex	*p* < 0.552	*p* < 0.522
Injection × Treatment	*p* < 0.001	*p* < 0.001
Sex × Treatment	*p* < 0.549	*p* < 0.668
Injection × Sex × Treatment	*p* < 0.947	*p* < 0.624

**Table 7:** Summary of two-way ANOVAs performed with data on protein levels of 4-HNE, NLRP3, and p-JNK in the DRG and AMG from CIS-injected male and female mice treated with VEH, DULO, HRW, or DULO plus HRW.

	DRG			AMG		
	4-HNE	NLRP3	p-JNK	4-HNE	NLRP3	p-JNK
Sex	*p* < 0.365	*p* < 0.757	*p* < 0.519	*p* < 0.468	*p* < 0.759	*p* < 0.062
Treatment	*p* < 0.004	*p* < 0.039	*p* < 0.002	*p* < 0.677	*p* < 0.001	*p* < 0.147
Sex × Treatment	*p* < 0.924	*p* < 0.675	*p* < 0.456	*p* < 0.834	*p* < 0.805	*p* < 0.614

**Table 8:** Summary of two-way ANOVAs performed with data on the relative protein levels of HO-1, SOD-1, and NQO1 in the DRG and AMG from CIS-injected male and female mice treated with VEH, DULO, HRW, or DULO plus HRW.

	DRG			AMG		
	HO-1	SOD-1	NQO1	HO-1	SOD-1	NQO1
Sex	*p* < 0.088	*p* < 0.827	*p* < 0.909	*p* < 0.054	*p* < 0.995	*p* < 0.359
Treatment	*p* < 0.001	*p* < 0.048	*p* < 0.118	*p* < 0.900	*p* < 0.907	*p* < 0.424
Sex × Treatment	*p* < 0.227	*p* < 0.856	*p* < 0.758	*p* < 0.892	*p* < 0.975	*p* < 0.895

## Data Availability

Data is contained within the article.

## Supplementary Materials

The following supporting information can be downloaded at: https://www.mdpi.com/article/10.3390/antiox14081004/s1.
