# Supplementary material for: Preventing Cisplatin-Induced Neuropathy and Related Emotional Disorders with the Coadministration of Duloxetine and Hydrogen-Rich Water in Male and Female Mice
Source: Antioxidants (Basel). 2025 Aug 16;14(8):1004. doi: 10.3390/antiox14081004 (PMC12383221; doi:10.3390/antiox14081004)
Supplement: Supplementary file 1 [file antioxidants-14-01004-s001.zip › antioxidants-3717389-supplementary.pdf]

The results of the AUC for the von Frey filaments strength (g), the number of hind paw lifts in the cold plate, the grip strength (g) and body weight (g) from male and female mice injected with VEH or CIS and treated with VEH, DULO and HRW alone and in combination for 30 consecutive days.

**GROUP**	**von Frey filaments strength (g)**		**Paw lifts (number)**		**Grip strength (g)**		**Body weight (g)**	
**SEX**	MALE	FEMALE	MALE	FEMALE	MALE	FEMALE	MALE	FEMALE
**VEH-VEH-VEH**	79.2 ± 2.7	80.1 ± 2.9	25.9 ± 3.7	31.6 ± 3.7	1866.0 ± 39.3	1669.0 ± 43.6	773.7 ± 23.8	672.8 ± 14.6
**VEH-DULO-VEH**	80.9 ± 2.6	81.0 ± 2.8	30.8 ± 3.8	29.2 ± 3.9	1874.5 ± 40.1	1624.4 ± 38.8	794.0 ± 24.1	696.6 ± 29.6
**VEH-VEH-HRW**	80.1 ± 2.7	80.1 ± 2.9	26.2 ± 3.7	28.8 ± 3.7	1838.3 ± 40.5	1616.0 ± 42.6	773.8 ± 23.1	681.1 ± 10.2
**VEH-DULO-HRW**	80.9 ± 2.6	81.3 ± 2.9	30.6 ± 3.6	29.5 ± 3.7	1902.5 ± 40.3	1620.8 ± 40.8	769.3 ± 23.4	699.7 ± 27.2
**CIS-VEH-VEH**	47.9 ± 2.7 *	50.7 ± 2.8 *	143.2 ± 4.6 *	179.0 ± 5.2 *	1512.3 ± 27.4 *	1249.1 ± 26.1 *	665.7 ± 15.1 *	541.7 ± 17.4 *
**CIS-DULO-VEH**	65.7 ± 2.7 *@	67.7 ± 2.9 *@	81.7 ± 4.6 *@	89.0 ± 4.5 *@	1775.0 ± 39.2 *@	1570.5 ± 39.2 @	712.8 ± 20.3	625.0 ± 14.5
**CIS-VEH-HRW**	63.7 ± 2.9 *@	66.1 ± 3.3 *@	108.0 ± 4.7 *@	112.5 ± 6.2 *@	1707.0 ± 38.1 *@	1500.0 ± 40.0 *@	728.0 ± 25.8	626.0 ± 24.1
**CIS-DULO-HRW**	78.2 ± 2.7 @+#	76.8 ± 2.9 @+#	53.6 ± 3.3 *@+#	73.6 ± 3.8 *@#	1850.4 ± 36.1 @#	1616.5 ± 45.3 @#	736.7 ± 22.3	621.4 ± 17.4

In all tests. symbols denote significant changes vs. their respective mice treated with VEH-VEH-VEH, VEH-DULO-VEH or VEH-VEH-HRW (*); CIS-VEH-VEH (@); CIS-DULO-VEH (+) or CIS-VEH-HRW (#) ($p < 0.05$. one-way ANOVA plus Holm-Šídák's multiple comparisons test). Mean values ± SEM; n = 8 animals
